# Genome-Wide Association Identifies *SLC2A9* and *NLN* Gene Regions as Associated with Entropion in Domestic Sheep

**DOI:** 10.1371/journal.pone.0128909

**Published:** 2015-06-22

**Authors:** Michelle R. Mousel, James O. Reynolds, Stephen N. White

**Affiliations:** 1 Range Sheep Production Efficiency Research Unit, Agricultural Research Service, Department of Agriculture, Dubois, ID, United States of America; 2 Animal Disease Research Unit, Agricultural Research Service, Department of Agriculture, Pullman, WA, United States of America; 3 Department of Veterinary Microbiology and Pathology, Washington State University, Pullman, WA, United States of America; China Agricultural Univeristy, CHINA

## Abstract

Entropion is an inward rolling of the eyelid allowing contact between the eyelashes and cornea that may lead to blindness if not corrected. Although many mammalian species, including humans and dogs, are afflicted by congenital entropion, no specific genes or gene regions related to development of entropion have been reported in any mammalian species to date. Entropion in domestic sheep is known to have a genetic component therefore, we used domestic sheep as a model system to identify genomic regions containing genes associated with entropion. A genome-wide association was conducted with congenital entropion in 998 Columbia, Polypay, and Rambouillet sheep genotyped with 50,000 SNP markers. Prevalence of entropion was 6.01%, with all breeds represented. Logistic regression was performed in PLINK with additive allelic, recessive, dominant, and genotypic inheritance models. Two genome-wide significant (empirical P<0.05) SNP were identified, specifically markers in *SLC2A9* (empirical P = 0.007; genotypic model) and near *NLN* (empirical P = 0.026; dominance model). Six additional genome-wide suggestive SNP (nominal P<1x10^-5^) were identified including markers in or near *PIK3CB* (P = 2.22x10^-6^; additive model), *KCNB1* (P = 2.93x10^-6^; dominance model), ZC3H12C (P = 3.25x10^-6^; genotypic model), JPH1 (P = 4.68x20^-6^; genotypic model), and *MYO3B* (P = 5.74x10^-6^; recessive model). This is the first report of specific gene regions associated with congenital entropion in any mammalian species, to our knowledge. Further, none of these genes have previously been associated with any eyelid traits. These results represent the first genome-wide analysis of gene regions associated with entropion and provide target regions for the development of sheep genetic markers for marker-assisted selection.

## Introduction

Entropion is an eye health problem where the eyelid rolls toward the eye, causing the hair and lashes to contact the eye and can cause blindness [1]. Blindness can occur because of corneal abrasion or secondary infections, which are common if the condition is not treated [2]. Several factors may contribute to the development of entropion, including congenital causes [3, 4], selective breeding [5, 6], scar tissue formation [7, 8], and/or age-related processes [9, 10]. Congenital entropion has been identified in numerous mammals, including humans [11], horses [12], cats [13], dogs [14], rabbits [15], pigs [16], cattle [17], goats [18], and sheep [1]. In dogs, entropion has been identified as an inherited disorder of high research priority, particularly in Chinese Shar-Pei which has a frequency of 14–60% and Bulldogs with a frequency of 58% [19]. The cost of surgery in the U.S. to correct entropion ranges from $300 to $1500 in dogs [20] and in humans is at least $3000 [21], therefore, entroprion correction in the identified species costs U.S. consumers at least tens of millions of dollars per year.

There is evidence of genetic components to the development of entropion in sheep [22]. Reported worldwide frequency of entropion in lambs is quite variable from 1.1% to 80% [1, 2, 4, 23, 24]. There are methods to correct entropion in lambs [25] but intervention by a veterinarian or trained personnel is costly, and untreated lambs may become blind [2]. Approximately 600,000 lambs in the U.S may be afflicted if entropion has a 10% frequency, costing about $150,000 per year to correct assuming a trained technician was paid minimum wage and could process 25 lambs per hour. These cost would increase significantly if a veterinarian was consulted for entropion correction. Heritability of entropion has been estimated to be 0.08–0.21 in Columbia, Polpay, Rambouillet, Suffolk, and Targhee breeds in the U.S. [22]. Because entropion is heritable, reduction of the condition within afflicted flocks can occur with careful selection when purchasing breeding sheep as well as culling the parents of and not breeding affected sheep. However, development of a genetic test for entropion would allow producers to more efficiently select replacement sheep without this defect.

Genotyping technologies exist which improve the probability of identifying genomic regions associated with phenotypic traits of interest in sheep. The Ovine SNP50 beadchip [26] was collaboratively, internationally developed and has been used to identify new markers associated with inherited diseases [27, 28], erythrocyte traits, [29], parasite infection [30], and other infectious disease traits [31, 32, 33]. We hypothesized that genomic regions related to development of entropion could be identified using genome-wide association using sheep as a model species. To ensure wide applicability of the findings and identify markers in more than 1 breed of sheep, 3 different breeds of sheep were evaluated in this study using genome-wide association with entropion.

## Materials and Methods

### Ethics Statement

All animal care and handling procedures were reviewed and approved by the Washington State University Institutional Animal Care and Use Committee (Permit Number: 3171) and/or by the U.S. Sheep Experiment Station Animal Care and Use Committee (Permit Numbers: 10–06, 10–07). All efforts were made to minimize discomfort during collection of blood samples.

### Populations and Phenotypes

At the U.S. Sheep Experiment Station (USSES), trained technicians determine entropion status within 48 hours of birth with entropion defined as the inward rolling of one or both lower eyelids in lambs. Upper eyelid entropion has never been documented is USSES lambs. All sheep born alive had entropion status recorded as either present or absent. Whole blood was collected from ewes of Rambouillet (N = 414), Polypay (N = 438), and Columbia (N = 146) breeds, ages 1–5 years. Entropion was documented in 60 of these ewes, with all breeds represented. These animals were managed in an extensive rangeland production system and bred in single sire mating pens.

### Genotyping

Blood was collected by jugular venipuncture into EDTA-coated vacutainer tubes. DNA was isolated using the Invitrogen GeneCatcher gDNA 3–10 ml Blood Kit as per manufacturers’ instructions (Life Technologies, Carlsbad, CA). The DNAs were checked for quality and quantity using an ND-1000 spectrophotometer (Nanodrop, Wilmington, DE) and equilibrated to 50ng/μl for genotyping. Genotyping services were provided by Geneseek Inc. (Lincoln, NE) using the OvineSNP50 Infinium BeadChip (Illumina Inc., San Diego, CA) with a set of 54,977 SNP designed by the International Sheep Genome Consortium [26].

### Association analysis

A preliminary screen for high genotype call rates (>97%) was performed to select individuals for further analysis. Multidimensional scaling analysis (MDS) of breed groups (S1 Fig [32]) and pairwise population concordance clustering were performed in PLINK v1.06 [34] as previously described [32]. Single nucleotide polymorphism inclusion screening criteria in PLINK analysis were as previously described [32]: missingness by individual (0.1), missingness by marker (0.03), minor allele frequency (0.01), and Hardy-Weinberg equilibrium (0.000001, which corresponds to P = 0.05 after Bonferroni correction for 50,000 marker tests). Four population groups were identified by population concordance clustering, including Polypay, Columbia, Rambouillet subgroup 1 and Rambouillet subgroup 2. The full model evaluated included fixed effects of population group (with 4 levels: Polypay, Columbia, Rambouillet subgroup 1, and Rambouillet subgroup 2) and SNP minor allele. Logistic regression was performed in PLINK to determine if there were genomic regions associated with presence or absence of entropion. An additional step assessed model fit for additive allelic, recessive, dominant, and genotypic inheritance models. Permutations were calculated within sire family as previously described [32]. Ten thousand permutations were performed to establish significance, and genome-wide significance was defined by empirical P<0.05. Genome-wide suggestive significance was defined by nominal P<1x10^-5^ per Wellcome Trust consortium guidelines [35]. PLINK only reports regression coefficients as a measure of effect size for linear regression, therefore, SAS 9.4 (SAS Institute, Cary, NC) was used to run similar genotypic models in the logistic procedure to obtain largest adjusted genotypic mean differences as a measure of effect size for entropion. An R script [36] provided by Dr. Stephen Turner (http://gettinggeneticsdone.blogspot.com/2011/04/annotated-manhattan-plots-and-qq-plots.html, viewed on 11-15-11) was used for visualization of results in Manhattan and quantile-quantile plots. Further, the top SNPs were interrogated using a threshold model similar to those described above, accounting for entropion status, breed/ population cluster and genotype in the probit procedure of SAS 9.4 (SAS Inst. Inc., Cary, NC). Ensembl was used to determine the location of the SNP within genome assembly OAR_v3.1 as well as identify Ensembl annotated genes within 100 kb of the SNP. S2 Table shows the Illumina designated name of the SNP with the dbSNP rs# cluster id.

## Results

The total number of sheep evaluated in this genome-wide association study was 964, reduced from the original 998 due to sample quality control criteria and breed outlier status. The number of ewes per breed represented were Rambouillet (n = 399), Polypay (n = 423), and Columbia (n = 142). These included 59 sheep with entropion where all breeds were represented. The average genotyping call rate was 98.06% for the final population of sheep evaluated.

**Fig 1:**
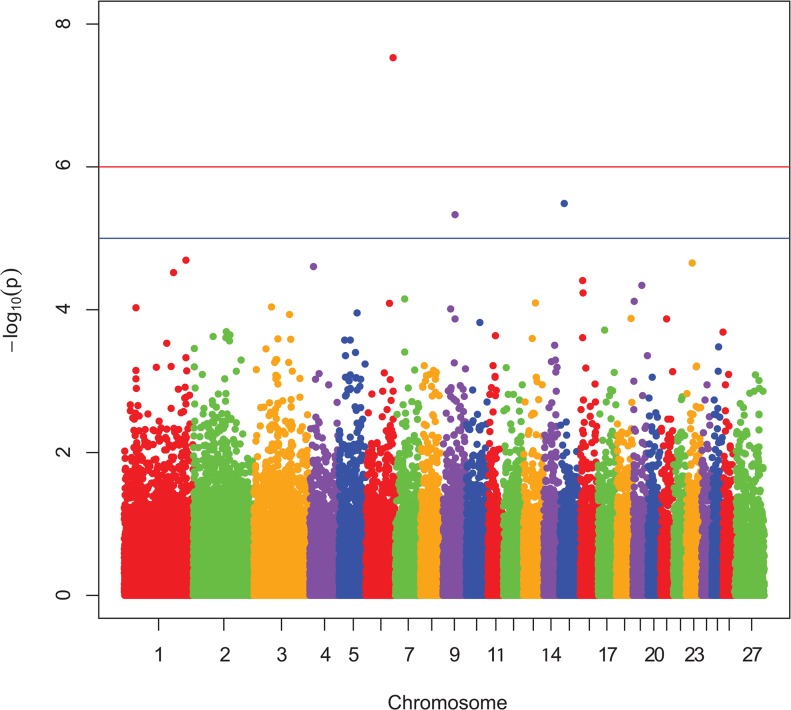
Genotypic Manhattan plot for entropion. The Manhattan plot shows nominal P-values from association with entropion by chromosomal position. Data from genotypic mode of inheritance analyses. The top red line shows a genome-wide significance threshold defined by nominal P-values of 1x10^-6^, which is P = 0.05/50,000. The lower blue line shows a genome-wide suggestive significance threshold (1x10^-5^).

**Fig 2:**
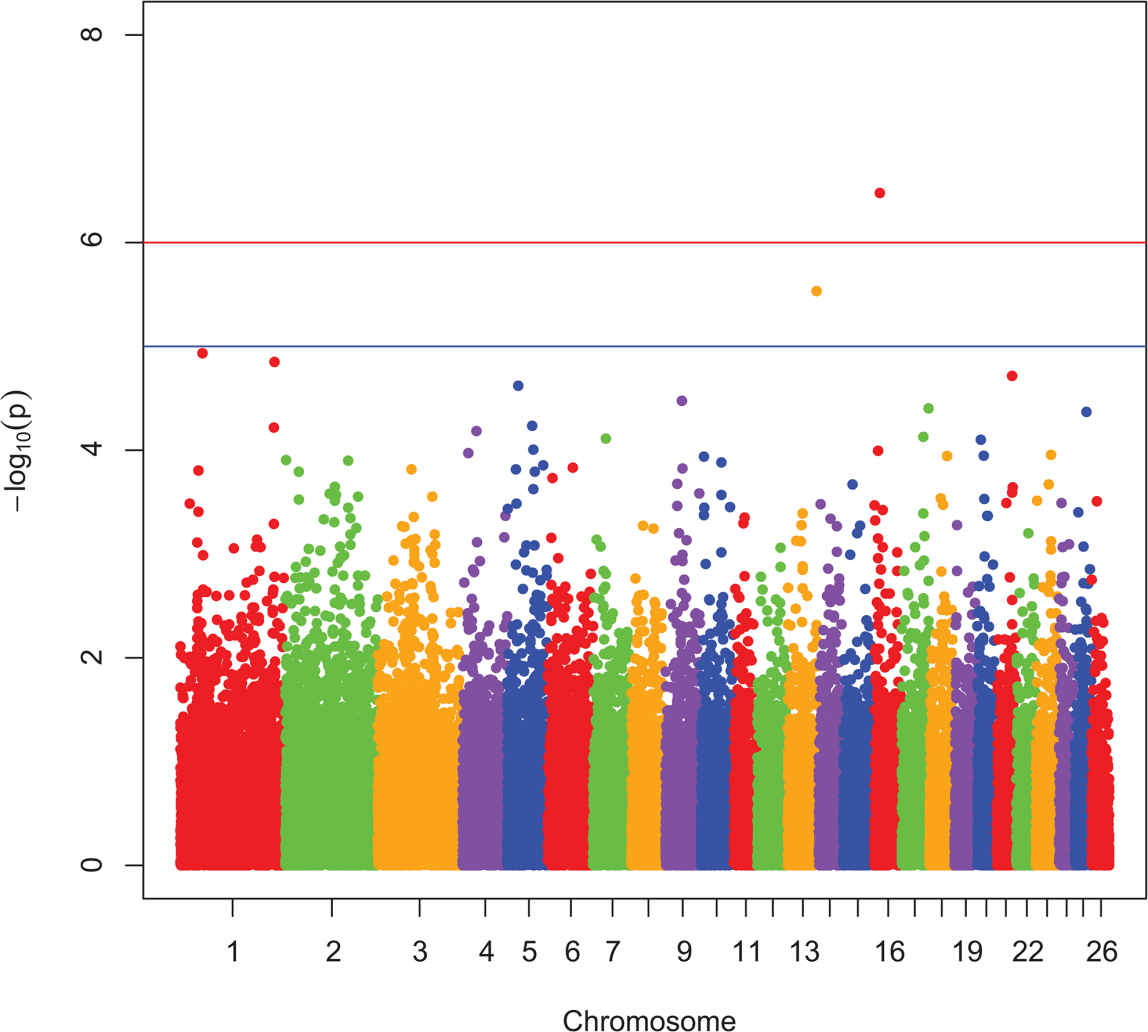
Dominant Manhattan plot for entropion. The Manhattan plot shows nominal P-values from association with entropion by chromosomal position. Data from dominant mode of inheritance analyses. The top red line shows a genome-wide significance threshold defined by nominal P-values of 1x10^-6^, which is P = 0.05/50,000. The lower blue line shows a genome-wide suggestive significance threshold (1x10^-5^).

**Fig 3:**
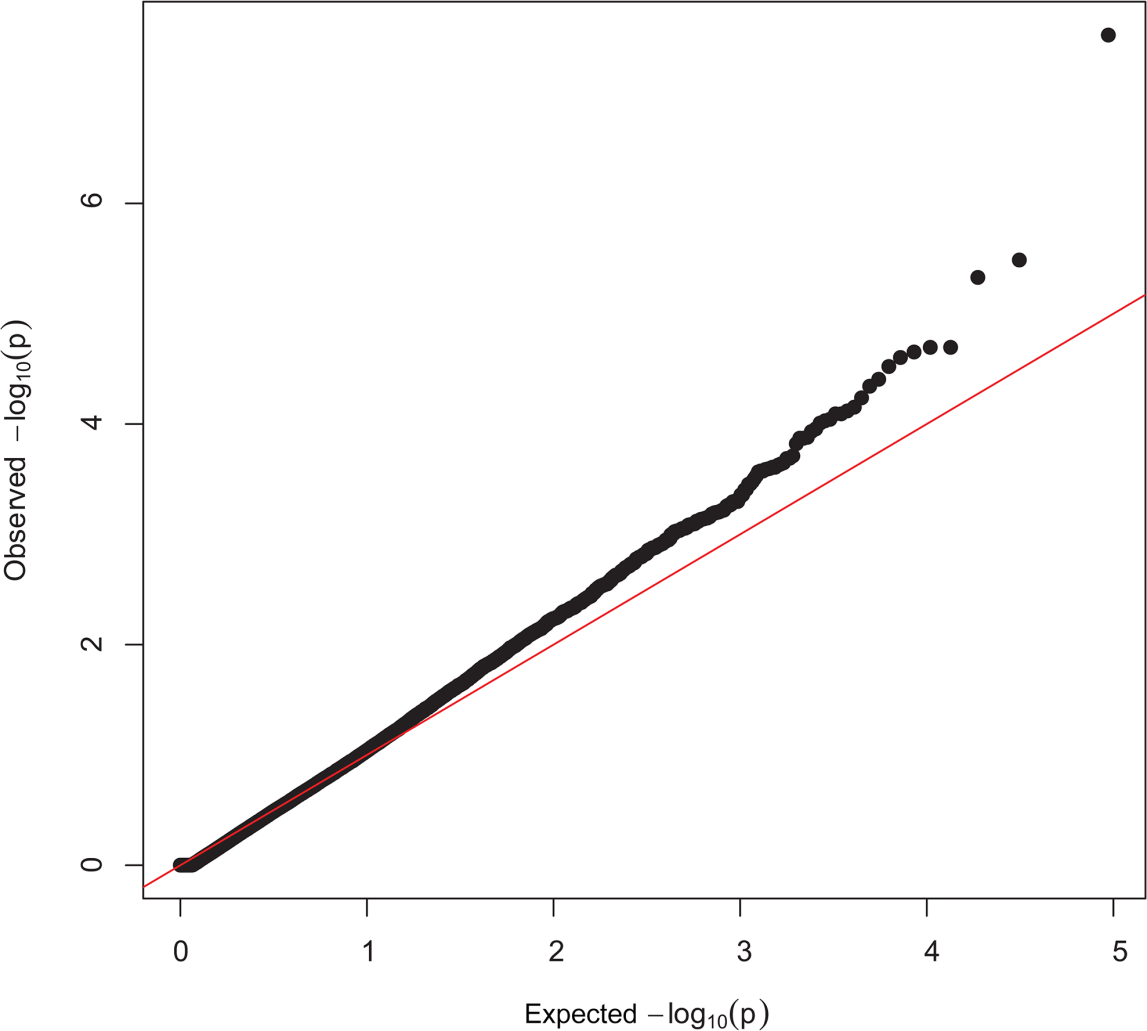
Quantile-quantile plots with all SNP from the genotypic analysis. Quantile-quantile plots from association with entropion, where the expected distribution is the red line.

**Fig 4:**
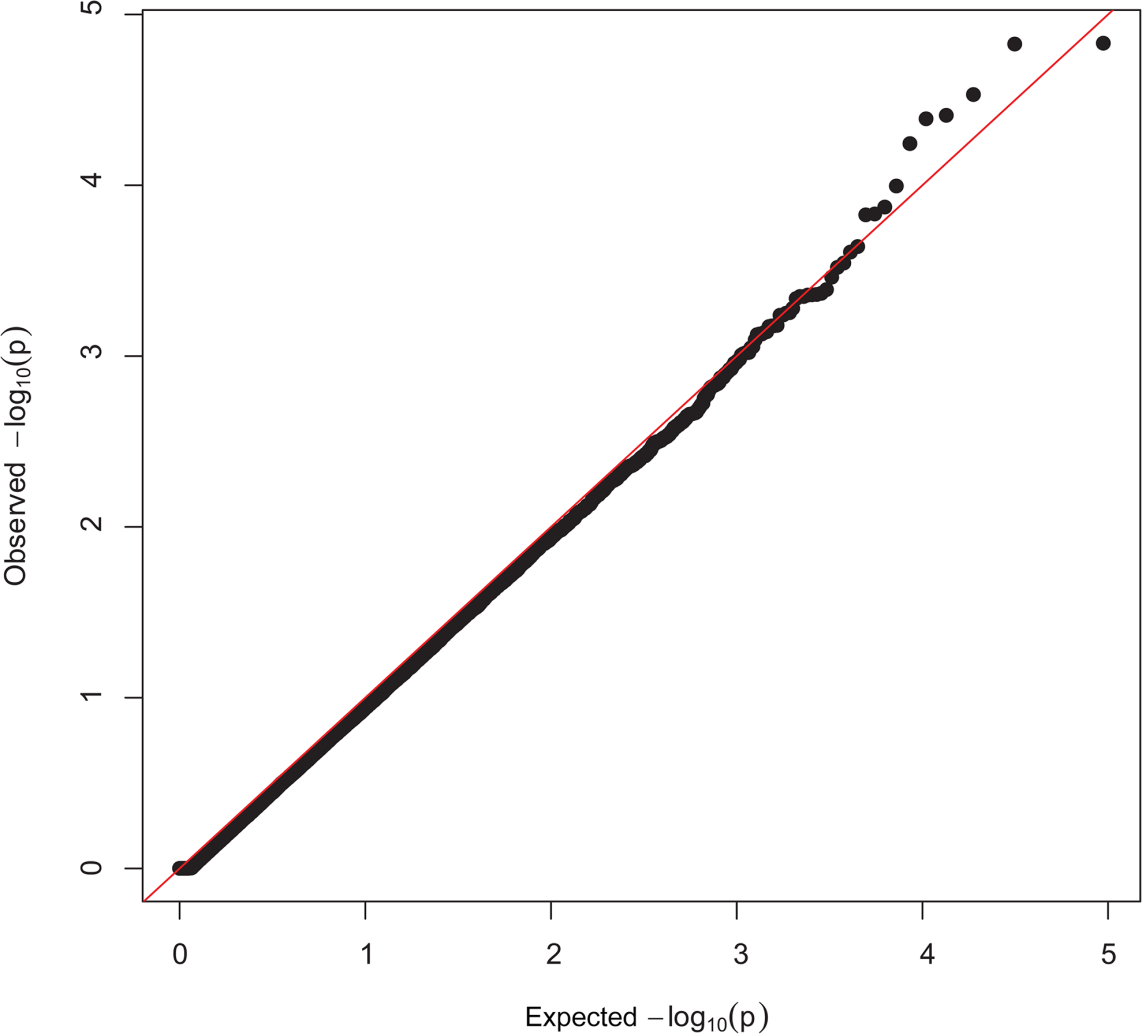
Quantile-quantile plots conditioned on 5 top SNP from the genotypic analysis. Quantile-quantile plots from association with entropion, where the expected distribution is the red line. Differences in mutation allele frequency were accounted for with 5 of the top SNP in the model which eliminated apparent population stratification.

Manhattan plots showing P-values in order of chromosome position, from the genotypic and dominant analysis, are presented in Fig 1 and Fig 2, respectively. Two SNP were classified as genome-wide significant and six SNP were genome-wide suggestive (Table 1). Observed vs expected P-value distributions were visualized in quantile-quantile plots from genotypic analyses with all SNP (Fig 3) and the conditioned on the following 5 SNP, rs424438792, rs420662001, rs420083564, rs419388939, and rs405483139, (Fig 4). Fig 3 shows some of the observed P-values were divergent from the expected line indicating possible population stratification, including potentially differing frequencies of a small number of genetic mutations. Nearly all stratification is eliminated after conditioning the analysis on the 5 SNP shown above (Fig 4). This demonstrates that the apparent population stratification was due to differences in allele frequencies of SNP associated with entropion. Dominant analysis quantile-quantile plots with all SNP and conditioned on the above 5 SNP are shown in S2 Fig and S3 Fig. The genotype count for the top 8 SNP by breed and entropion status are shown in S1 Table and the raw data for these SNP may be found at: http://www.ncbi.nlm.nih.gov/projects/SNP/snp_viewBatch.cgi?ibid=1062140 or http://www.animalgenome.org/repository/pub/USDA2015.0208/ Threshold analysis showed rs424438792, rs720662001, rs420083564, rs703034846, rs419388939, and rs405483139 significantly (*P <* 0.04) and rs415069937 and rs401620279 were not (*P >* 0.06) associated with entropion. The sheep genome assemble Oar_v3.1 [26] was used to determine the location of the top 8 SNP associated with entropion which were found to be located within or near the following genes: solute carrier family 2 C 9 (*SLC2A9*, a.k.a. GLUT9), phosphatidylinositol 4,5-bisphospate 3-kinase, catalytic subunit beta isoform (*PIK3CB*), myosin-IIIb (*MYO3B*), potassium voltage-gated channel subfamily B member 1 (*KCNB1*), neurolysin (*NLN*), zinc finger CCCH-type containing 12C (*ZC3H12C*), and junctophilin 1 (*JPH1*).

**Table 1:** Genomic regions associated with entropion.

*SNP*	*Chr*	*Position (bp)*	*Best fitting model*	*Nominal P-value*	*Empirical P-value*	*Odds Ratio*	*Genes within 100 Kb on either side*
rs424438792	6	104,687,454	genotypic	2.97x10^-8^	0.007	41.77	SLC2A9**
rs420662001	16	13,738,722	dominant	3.34x10^-7^	0.026	11.01	NLN*, ERBB2IP
rs420083564	1	248,360,688	additive	2.22x10^-6^	^§^	3.18	PIK3CB**, PRR23B
rs403034846	1	248,348,219	additive	2.22x10^-6^	^§^	3.18	PIK3CB*, PRR23B
rs419388939	13	77,135,671	dominant	2.93x10^-6^	^§^	4.06	KCNB1**, PTGIS
rs415069937	15	19,548,621	genotypic	3.25x10^-6^	^§^	12.26	ZC3H12C
rs401620279	9	50,318,215	genotypic	4.68x10^-6^	^§^	7.82	JPH1*
rs405483139	2	138,159,566	recessive	5.74x10^-6^	^§^	4.27	MYO3B**

^§^: P>0.15

**: SNP located within gene

*: SNP located within 35 Kb of gene

## Discussion

Eyelid development in embryonic mammals has long been known to consist of four primary stages: specification, growth, epithelial fusion, and reopening [37, 38]. Many genes have been identified in these developmental stages using multiple phenotypes in mice including eyes-open-at-birth (EOB), and these have been grouped into two major pathways: Activin-MEKK1-JNK and TGFalpha-EGFR-ERK [38]. These pathways are required for correct eyelid development from both epithelial and mesenchymal cell layers and are important in coordinating interactions between the two [38].

However, much less is known about causes of the entropion, and especially about congenital entropion. Studies of entropion with a large infectious disease component (trachomatous entropion) have implicated contraction of the subepithelial fibrous membrane, which is formed by vertically oriented parallel collagen fibers [39]. Age-related entropion has been shown to be due largely to differences in the size of tarsal plates, the dense fibrous connective tissue that gives support and shape to the eyelid [40]. While less is known about the causes of congenital entropion, both tarsal plate problems and muscular hypertrophy have been implicated [41]. Together these results suggest fibroblasts, keratinocytes, macrophages, and myocytes, among others, may be key cell types for genetic influence on incidence of entropion [42, 43].

This is the first genome-wide association study to identify gene regions for any form of entropion in mammals, to our knowledge, and we identified two genome-wide significant and 5 genome-wide suggestive regions. None of the genes associated with the identified SNP were found directly within the Activin-MEKK1-JNK or TGFalpha-EGFR-ERK pathways [38].

The first genome-wide significant SNP (s65132) was located on chromosome 6 within *SLC2A9*. This SNP was identified in both the recessive and genotypic analysis, the genotypic model had both the smallest P-value (empirically P = 0.007) and the largest odds ratio (41.77) of any SNP in this study. The odds ratio indicated a large difference in odds of entropion by genotype, well above the odds ratio of 2 commonly used as a threshold for large effect size [44]. The *SLC2A9* gene encodes a glucose, fructose, and urate transporter with alternate splice isoforms expressed on different surfaces of polarized cells [45, 46]. In some tissues, the GLUT9 protein product of the *SLC2A9* gene is a major contributor to glucose influx [47]. Expression of *SLC2A9/GLUT9* has been shown in muscle cells at the mRNA and protein levels, and the GLUT9 protein may play a role in muscle cell proliferation [48]. Thus, it is possible that *SLC2A9* could be involved in muscle tone contributions to the development of entropion. While the *SLC2A9* gene has been most frequently associated with gout and renal disease in humans [49, 50], this is the first report of *SLC2A9* in connection to eyelid shape and positioning.

The chromosome 16 genome-wide significant SNP (OAR16_14874751), identified in both the additive and dominant analysis, had a large odds ratio of 11.01 in the dominant analysis, and was within 35Kb of *NLN* in version 3.1 of the ovine genome [51]. Neurolysin is a member of the metallopepidase M3 protein family that cleave many substrates including neurotensin and may play a role in the termination of the neurotensinergic signal in the central nervous system [52]. Neurolysin knock-out mice demonstrated that this enzyme plays a role in energy metabolism with mRNA expression differences in liver and overall increased glucose tolerance, insulin sensitivity, and gluconeogenesis [53]. In addition, NLN is part of the angiotensin-(1–7), MAS1 pathway in which genetic mutations could impact cellular development [54]. Further, active neurotensin (cleaved by neurolysin) increases epidermal growth factor expression [55], which connects NLN to the TGFalpha-EGFR-ERK pathway important for eyelid development [38]. Interestingly, neurotensin also modulates macrophage migration and inflammatory response under hyperglycemic conditions [56]. Since macrophages are one of the major cell types within eyelids [42], it is possible that *SLC2A9* might be functionally related to *NLN* in the development of entropion through the influence of sugar transport on neurotensin activity.

The other 6 genomic loci identified in this study were genome-wide suggestive. Specifically on chromosome 1, the two suggestive SNP were adjacent to one another on the Ovine SNP50 beadchip, were counted as one genomic locus, and were in the top 5 SNP of the additive and dominant analysis. This locus encompassed *PIK3CB* which is found on the outer membrane of eukaryotic cells and is important in many cellular pathways [57]. *PIK3CB* may modulate cell morphology [57], cell division, cell motility, metabolism, and apoptosis [58], thus potentially impacting neonatal development of entropion. In addition, p85α, a subunit of *PIK3*, has been shown to be required for mesenchymal stem cells to differentiate into osteoblasts, adipocytes, and chondrocytes *in vitro* [59]. The chromosome 2 genome-suggestive SNP, identified in the recessive analysis, was located within *MYO3B* which is a protein that mediates movement along actin filaments in the cell [60]. A GWAS evaluating adipose growth and deposition indicated that *MYO3B* may play a role in adipose deposition [61]. In context of this study, less adipose in the eyelid during development may play a role in entropion. The chromosome 13 genome-suggestive SNP, identified in the top 5 SNPs of both the additive and dominant analysis, was positioned within *KCNB1* which plays a role in apoptosis in neurons [62] and may impact cell volume [63]. Cells with less volume within the eyelid may lead to entropion. The chromosome 15 suggestive SNP, identified in both additive and dominant analysis, had a large odds ratio of 12.26, and was within 100kb of *ZC3H12C*. This recently identified gene has been found to inhibit inflammation in vitro [64]. The chromosome 9 genome-suggestive SNP, located within 35 kb of *JPH1*, was identified in the top 5 SNPs of both the additive and dominant analysis. This gene is expressed in skeletal muscle and plays a role in intramembrane Ca^2+^ movement [65]. Reduced expression of *JPH1* has been shown in inactive, weak skeletal muscle [66]. If the muscles underlying the eye lid were weak, this could conceivably lead to entropion.

In conclusion, we undertook the first genome-wide association study of entropion in any mammal to provide insight into genes and mechanisms influencing this condition. This research identified five chromosomal regions that were associated with entropion in three common U.S. sheep breeds, including two that were associated with very large odds ratios. Several genes have logical involvement in the development of entropion, but none had been implicated in eyelid traits previously. These results provide insight into the development of entropion in mammals, and they provide target genes and genomic regions for mutation discovery, which is ongoing. Furthermore, identifying and validating one or more markers for marker-assisted selection from future work in these genomic regions would help producers to reduce the incidence of entropion within their sheep flocks and potentially improve production.

## Supporting Information

S1 Fig MDS cluster plot. Columbia are included in the top cluster, Polypay in the bottom right cluster, and Rambouillet in the bottom left cluster. The clustering of individuals by breed is clear even from these related breeds. This also shows how the Rambouilllet were split into 2 groups with the small cluster above and to the right of the main cluster [32].
(PDF)

S2 Fig Quantile-quantile plots with all SNP from the dominant analysis. Quantile-quantile plots from association with entropion, where the expected distribution is the red line.
(EPS)

S3 Fig Quantile-quantile plots condition on 5 top SNP from the dominant analysis. Quantile-quantile plots from association with entropion, where the expected distribution is the red line.
(EPS)

S1 Table Genotype count for the top 8 SNP by breed and entropion status. (DOCX)

S2 Table Illumina designated SNP name with reference SNP (rs) number. (DOCX)
