# Supplementary figures and images for: Genome-Wide Association Identifies SLC2A9 and NLN Gene Regions as Associated with Entropion in Domestic Sheep
Source: PLoS One. 2015 Jun 22;10(6):e0128909. doi: 10.1371/journal.pone.0128909 (PMC4476619; doi:10.1371/journal.pone.0128909)

**Figure S2: Multidimensional Scaling after Screening Outliers**

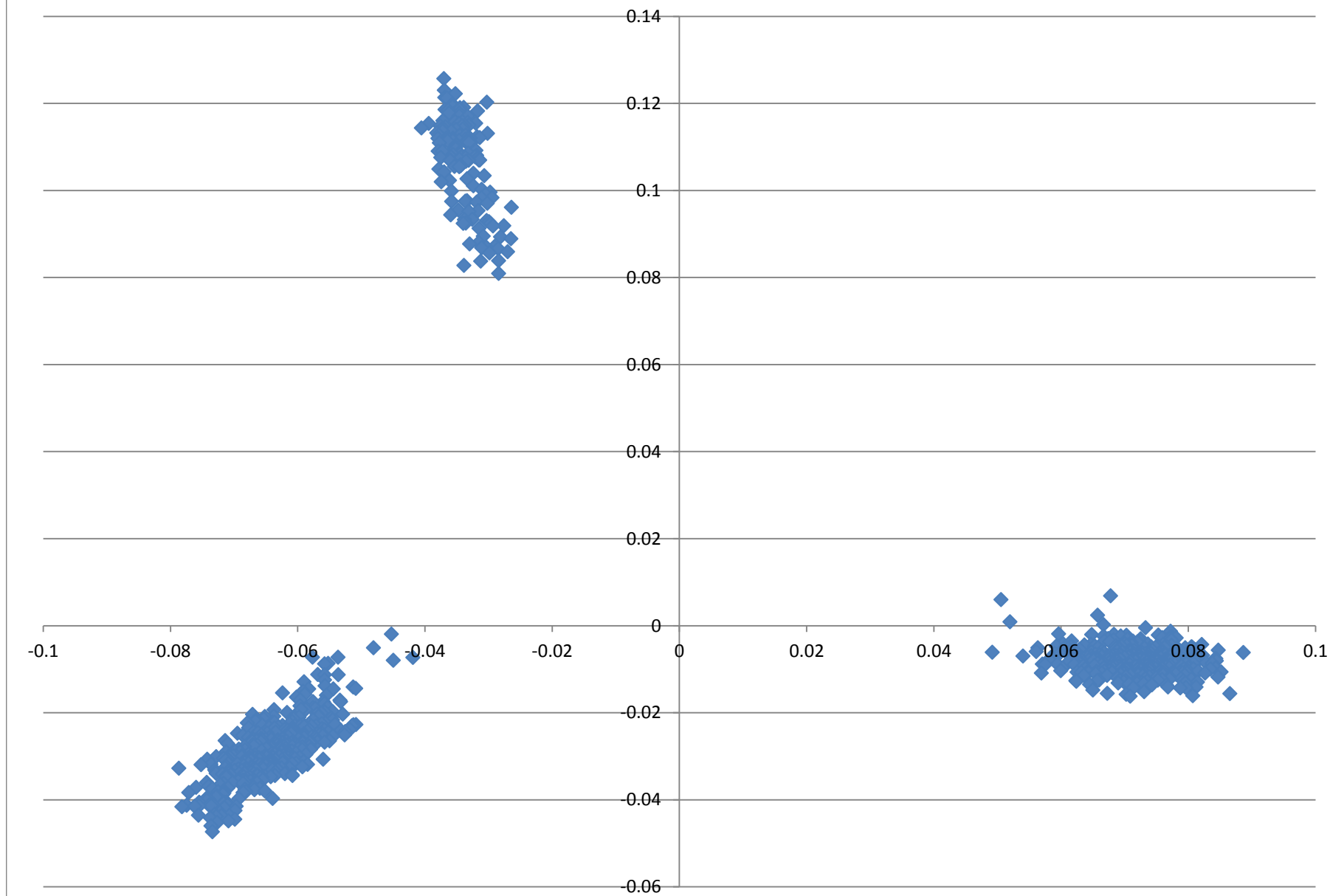

Supplement: S1 Fig — Columbia are included in the top cluster, Polypay in the bottom right cluster, and Rambouillet in the bottom left cluster. The clustering of individuals by breed is clear even from these related breeds. This also shows how the Rambouilllet were split into 2 groups with the small cluster above and to the right of the main cluster [32]. (PDF) [file pone.0128909.s001.pdf]
