# Supplementary material for: Genome-Wide Association Identifies SLC2A9 and NLN Gene Regions as Associated with Entropion in Domestic Sheep
Source: PLoS One. 2015 Jun 22;10(6):e0128909. doi: 10.1371/journal.pone.0128909 (PMC4476619; doi:10.1371/journal.pone.0128909)
Supplement: S1 Table — (DOCX) [file pone.0128909.s004.docx]

**Table S1 – Genotype count for the top 8 SNP by breed and entropion status**

| *SNP* | *Breed* | *Entropion*  *Status* | *Allele*  *1* | *Allele*  *2* | *Homozygous*  *1* | *Heterozygous* | *Homozygous*  *2* |
| --- | --- | --- | --- | --- | --- | --- | --- |
| rs424438792 | Columbia | Yes | C | T | 28 | 5 | 0 |
|  | Polypay | Yes | C | T | 3 | 1 | 1 |
|  | Rambouillet | Yes | C | T | 13 | 4 | 4 |
|  | Columbia | No | C | T | 99 | 10 | 0 |
|  | Polypay | No | C | T | 323 | 92 | 3 |
|  | Rambouillet | No | C | T | 294 | 80 | 4 |
| rs20662001 | Columbia | Yes | A | G | 0 | 4 | 29 |
|  | Polypay | Yes | A | G | 0 | 1 | 4 |
|  | Rambouillet | Yes | A | G | 0 | 6 | 15 |
|  | Columbia | No | A | G | 0 | 1 | 108 |
|  | Polypay | No | A | G | 1 | 46 | 371 |
|  | Rambouillet | No | A | G | 0 | 14 | 364 |
| rs420083564 | Columbia | Yes | A | G | 12 | 20 | 1 |
|  | Polypay | Yes | A | G | 2 | 2 | 1 |
|  | Rambouillet | Yes | A | G | 3 | 10 | 8 |
|  | Columbia | No | A | G | 79 | 29 | 1 |
|  | Polypay | No | A | G | 202 | 181 | 35 |
|  | Rambouillet | No | A | G | 132 | 176 | 70 |
| rs403034846 | Columbia | Yes | A | G | 12 | 20 | 1 |
|  | Polypay | Yes | A | G | 2 | 2 | 1 |
|  | Rambouillet | Yes | A | G | 3 | 10 | 8 |
|  | Columbia | No | A | G | 79 | 29 | 1 |
|  | Polypay | No | A | G | 202 | 181 | 35 |
|  | Rambouillet | No | A | G | 132 | 176 | 70 |
| rs401620279 | Columbia | Yes | C | T | 1 | 13 | 19 |
|  | Polypay | Yes | C | T | 0 | 2 | 3 |
|  | Rambouillet | Yes | C | T | 2 | 11 | 8 |
|  | Columbia | No | C | T | 1 | 21 | 87 |
|  | Polypay | No | C | T | 6 | 94 | 318 |
|  | Rambouillet | No | C | T | 4 | 62 | 312 |
| rs415069937 | Columbia | Yes | C | T | 0 | 6 | 27 |
|  | Polypay | Yes | C | T | 3 | 1 | 1 |

| Rambouillet | Yes | C | T | 5 | 10 | 6 |
| --- | --- | --- | --- | --- | --- | --- |
| Columbia | No | C | T | 5 | 63 | 41 |
| Polypay | No | C | T | 233 | 162 | 23 |
| Rambouillet | No | C | T | 111 | 195 | 72 |
| rs401620279 Columbia | Yes | C | G | 21 | 11 | 1 |
| Polypay | Yes | A | G | 3 | 1 | 1 |
| Rambouillet | Yes | A | G | 8 | 7 | 6 |
| Columbia | No | A | G | 95 | 14 | 0 |
| Polypay | No | A | G | 215 | 181 | 22 |
| Rambouillet | No | A | G | 211 | 150 | 17 |
| rs405483139 Columbia | Yes | C | T | 13 | 13 | 7 |
| Polypay | Yes | C | T | 2 | 1 | 2 |
| Rambouillet | Yes | C | T | 4 | 12 | 5 |
| Columbia | No | C | T | 72 | 35 | 2 |
| Polypay | No | C | T | 141 | 218 | 59 |
| Rambouillet | No | C | T | 149 | 185 | 44 |
