# Supplementary material for: Genome-Wide Association Identifies SLC2A9 and NLN Gene Regions as Associated with Entropion in Domestic Sheep
Source: PLoS One. 2015 Jun 22;10(6):e0128909. doi: 10.1371/journal.pone.0128909 (PMC4476619; doi:10.1371/journal.pone.0128909)
Supplement: S2 Table — (DOCX) [file pone.0128909.s005.docx]

**Table S2 - Illumina designated SNP name with reference SNP (rs) number.**

| rs number | Illuminia SNP name |
| --- | --- |
| rs424438792 | s65132 |
| rs420662001 | OAR16_14874751 |
| rs420083564 | OAR1_268186998 |
| rs403034846 | OAR1_268175642 |
| rs419388939 | s63760 |
| rs415069937 | OAR2_146760496 |
| rs401620279 | s45649 |
| rs405483139 | s71875 |
